# A Combination of Cervicovaginal Fluid Glutamate, Acetate and D-Lactate Identified Asymptomatic Low-Risk Women Destined to Deliver Preterm: a Prospective Cohort Study

**DOI:** 10.1007/s43032-021-00711-2

**Published:** 2021-08-10

**Authors:** Emmanuel Amabebe, Dilly O. C. Anumba

**Affiliations:** 1 Department of Oncology and Metabolism, University of Sheffield, Sheffield, UK; 2 Academic Unit of Reproductive and Developmental Medicine-Obstetrics and Gynaecology, Department of Oncology and Metabolism, University of Sheffield, 4th Floor, Jessop Wing, Tree Root Walk, Sheffield, S10 2SF UK

**Keywords:** Preterm birth, Cervicovaginal fluid, Metabolites, Asymptomatic low-risk women

## Abstract

Due to the modest predictive capacities and limited clinical application of transvaginal ultrasonographic cervical length (CL) and quantitative fetal fibronectin (qfFN) in pregnant women at low risk of preterm birth (PTB), we sought to determine the utility of cervicovaginal fluid (CVF) metabolites (by-products of host-microbial metabolism) for prediction of spontaneous PTB in asymptomatic low-risk women at mid-gestation. This was a prospective sub-cohort study from the ECCLIPPx study cohort. CVF from asymptomatic singleton women (20–22 weeks, *n* = 168) without a prior history of PTB were analysed for metabolites by enzyme-based spectrophotometry. CL, vaginal pH and qfFN were also measured. Correlation and predictive analyses were performed by Spearman’s correlation, and binary logistic regression and area under receiver operating characteristic curve (AUC), respectively. Of the 168 women enrolled, only CVF samples from 135 (80.4%) women were analysed. There were 6/135 (4.4%) spontaneous PTB (sPTBs), with two of these pregnancies ending ≤ 28 weeks’ gestation. Individually (AUC, 95% CI), only glutamate (0.72, 0.64–0.80) and CL (0.69, 0.60–0.77) were predictive of PTB. However, five multivariable models that more accurately predicted sPTB were also identified, i.e. a combination of: glutamate, acetate and D-lactate (GAD, 0.82, 0.74–0.89); CL and qfFN only (0.78, 0.70–0.85); CL, qfFN, glutamate and acetate (0.88, 0.81–0.93); CL, qfFN and GAD (0.94, 0.88–0.98); and GAD and pH (0.86, 0.79–0.92). Correlations between CL, pH and qfFN and metabolites were also observed. In this cohort, a midtrimester combination of CVF glutamate, acetate and D-lactate predicted preterm birth more accurately than individual metabolites, cervical length and fetal fibronectin with a very low false-positive rate and high positive predictive value. Further testing in populations with higher preterm birth rates is required.

**Supplementary Information:**

The online version contains supplementary material available at 10.1007/s43032-021-00711-2.

## Introduction

Accurate prediction of deliveries before 37 completed weeks of gestation (preterm birth, PTB) in asymptomatic pregnant women with transvaginal ultrasound cervical length (CL) > 25 mm and without a prior history of PTB has been a challenge to obstetricians and other caregivers. Such women are reported to have a lower risk of spontaneous PTB (sPTB) compared to women with a previous history of PTB and short cervix [1, 2]. Women with short cervix ≤ 25 mm estimated by transvaginal ultrasound at mid-trimester (18–24 weeks) have a 4–5 times greater risk of sPTB than women with normal cervical length (> 25 mm) [3–5]. A mid-trimester short cervix < 15 mm is associated with a 34% increased risk of PTB [2, 6]. A previous history of PTB increases a woman’s risk of subsequent premature deliveries by 4–6 times [2, 7]. This risk is often aggravated in the presence of a short cervix though such women may benefit from antenatal progesterone treatment [2, 8].

Currently employed diagnostic tools for determining a woman’s risk of PTB such as mid-trimester ultrasound cervical length and quantitative cervicovaginal fetal fibronectin (qfFN) measurements have limited predictive utility in asymptomatic low risk women [2, 9–11]. Although they show moderate predictive capacities in asymptomatic high-risk women [2, 12–14], routine screening of all asymptomatic pregnant women (irrespective of risk status) at mid-gestation with these tools have been controversial [2, 11, 15, 16], hence, not widely recommended [17–19]. This controversy is further compounded by their unresolved cost effectiveness [9].

Recently, in a bid to discover novel risk assessment strategies to guide preventive interventions for sPTB, our group reported that mid-trimester cervical electrical impedance spectroscopic (EIS) assessment predicts sPTB in a cohort comprising of both asymptomatic high- and low-risk women, with an improvement in the predictive accuracy when previous history of PTB was incorporated into the model [12]. This model was more predictive of sPTB than transvaginal ultrasound CL and qfFN individually. The predictive accuracy of EIS for sPTB was not improved by the addition of CL and qfFN [12].

Due to the modest predictive capacities of CL and qfFN measurements in asymptomatic women without a previous history of PTB [1, 2, 14, 20–22], other approaches to predicting PTB in this group require investigation. Such approaches include investigation of biomarkers that result from cervicovaginal host-microbial interactions which lead to dysbiosis and subclinical infection [2]. For instance, lactate, glutamate and acetate are markers of host-microbial interactions within the cervicovaginal space. Elevated D-lactate and glutamate are associated with eubiosis, whereas an increase in acetate and other short chain fatty acids (SCFAs) are associated with dysbiosis and infection [23]. Furthermore, increased lactate [24] and acetate [25–27] have opposite implications for eventual delivery outcome. Therefore, we sought to determine the utility of cervicovaginal fluid (CVF) metabolites, which are by-products of host-microbial metabolism, for prediction of sPTB in asymptomatic low-risk women at mid-gestation. We also aimed to test the relationship between CL, qfFN, vaginal pH and CVF metabolite concentrations in this unique cohort of pregnant women.

## Method

This was a prospective sub-cohort study from the ECCLIPPx study cohort [12] approved by the Yorkshire and Humber (Sheffield) Committee of the UK National Research Ethics Service (REC Number 13/YH/0167). The study participants were 168 asymptomatic singleton women at 20–22 weeks of gestation without a prior history of PTB and cervical length > 25 mm on transvaginal ultrasonography. These women were considered as being at low risk of delivering prematurely [1, 26], and were enrolled into the study between May 2013 and September 2015 after written informed consent was obtained. The study was conducted at the fetomaternal unit of the Jessop Wing Maternity branch of the Royal Hallamshire Hospital, Sheffield, UK, a tertiary PTB referral centre with around 7500 births annually. The eligibility and exclusion criteria are listed in Table 1. PTB outcome was defined as spontaneous delivery before 37 completed weeks of gestation. Cervicovaginal fluid samples were obtained prior to any vaginal examination or clinical intervention.

**Table 1:** Eligibility and exclusion criteria

Eligibility	Gestational age 20–22 weeks No prior PTB Cervical length > 25 mm Singleton gestation No symptoms of preterm labour Intact fetal membranes
Exclusion	Prior history of PTB Cervical length < 25 mm Symptoms suggestive of preterm labour PPROM Genital tract infection Urinary tract infection Abnormal cervical cytology Multiple gestation Recent vaginal examination Vaginal bleeding Fetal anomaly Cervical cerclage

*PPROM*, preterm prelabour rupture of membranes; *PTB*, preterm birth

## CVF Sample Collection and Preparation

A sterile Cusco’s vaginal speculum was passed and two high swabs were obtained from the posterior vaginal fornix of each pregnant woman with sterile Dacron swab (Deltalab Eurotubo 300,263, Fisher Scientific, UK) at presentation. The swabs were processed as previously described [24–27]. Briefly, immediately after collection, swabs were stored at − 20 °C and processed within 1–3 days, by placing in a 1.5-μL microfuge tube containing 600 μL isotonic phosphate buffered saline (PBS). The microfuge tube containing the cut end of the swab suspended in PBS was vortexed for 5 min to wash the CVF into solution. The swab tip was safely discarded, and the remaining solution was centrifuged for 3 min at 13,000 rpm after which the supernatant was aspirated into a fresh tube and preserved at − 80 °C until further analysis. Processing CVF samples immediately or storage in either − 20 °C or later in − 80 °C before metabolomic analysis does not usually affect metabolite concentrations [28].

Additionally, transvaginal ultrasound cervical length (CL), quantitative CVF fetal fibronectin (qfFN, 10Q Rapid analyser, Hologic, MA, USA) and vaginal pH (narrow range pH paper—pH-Fix 3.6–6.1, Machery-Nagel, Düren, Germany) [29] were also measured immediately after CVF sample was obtained. All samples and measurements were obtained by a single fully trained clinical operator.

## CVF Metabolite Measurement

CVF metabolite concentrations were measured by enzyme-based spectrophotometry on the ChemWell® 2910 auto-analyser (Awareness Technology, USA) using specific metabolite assay kits: acetate (K-ACETGK 08/14), glutamate (K-GLUT 07/12), L- and D-lactate (K-LATE 07/14 and K-DATE 04/14), glucose (K-GLUHK-110A/K-GLUHK-220A 07/14), formate (K-FORM 10/20) and succinate (K-SUCC 01/14) sourced from Megazyme (Cork, IE) and employed according to manufacturer’s instructions. These assay kits have been employed successfully in previous similar metabolomic analyses [25, 27, 30].

### Data analysis

The clinical and demographic characteristics as well as CVF metabolite concentrations of women with preterm and term delivery were subjected to Shapiro–Wilk normality test before analysis by Mann–Whitney *U* test or unpaired Student’s *t*-test depending on the result of the normality test. The strength and direction of associations between CVF metabolite concentrations and clinical and demographic characteristics of study participants were determined by Spearman’s rank-order correlation coefficient (*rho*). Predictive analyses were performed by binary logistic regression and area under receiver operating characteristic curve (AUC). Probability (*P*) values < 0.05 were considered statistically significant. All analyses were performed using GraphPad Prism 8.2 (GraphPad Software, Inc. USA), and MedCalc 20.0 (MedCalc Software bvba, Ostend, BE; http://www.medcalc.org; 2021) statistical software packages.

## Results

Maternal clinical and demographic details are shown in Table 2. Of the 168 predominantly Caucasian women enrolled in the study, only CVF samples from 135 (80.4%) women were analysed for metabolites by enzyme-based spectrophotometry—a targeted metabolomics technique with great potential for clinical translation. Data from 33 women were not included in further analysis due to lack of consent, loss to follow up, e.g. delivery in another facility, and the stringent eligibility/exclusion criteria listed in Table 1. There were 6/135 (4.4%) spontaneous births before 37 weeks’ gestation, with two of these pregnancies ending very preterm (i.e. ≤ 28 weeks’ gestation). There was an 8 weeks difference in mean gestational age at delivery (GAAD) between the term and preterm women with the average GAAD for the preterm-delivering women being < 32 weeks. Apart from the birth outcomes, no other recorded clinical or demographic characteristic differed significantly between the two groups of pregnant women.

**Table 2:** Maternal clinical and demographic characteristics according to birth outcome

Characteristic	Term (*n* = 129)	Preterm (*n* = 6)	*P* value
Age, years	28.93 ± 5.07 (*n* = 127)	28.33 ± 6.09 (*n* = 6)	0.78
BMI, kg/m^2^	25.34 ± 4.55 (*n* = 126)	23.76 ± 2.22 (*n* = 5)	0.55
Vaginal pH	3.98 ± 0.46 (*n* = 128)	4.08 ± 0.44 (*n* = 6)	0.72
qfFN, ng/mL	15.84 ± 34.74 (*n* = 127)	5.17 ± 3.49 (*n* = 6)	0.59
Cervical length, mm	40.27 ± 6.19 (*n* = 129)	36.67 ± 3.39 (*n* = 6)	0.16
GAAP, weeks	19.84 ± 0.83 (*n* = 129)	20.00 ± 0.63 (*n* = 6)	0.49
GAAD, weeks	39.78 ± 1.33 (*n* = 128)	31.67 ± 3.83 (*n* = 6)	** < 0.0001**

Data are presented as mean ± standard deviation

The reduced study population (*n*) in some parameters are due to absence of participant’s consent and/or data

*BMI*, body mass index; *GAAD*, gestational age at delivery; *GAAP*, gestational age at presentation/sampling; *qfFN*, quantitative fetal fibronectin

**Fig. 1:**
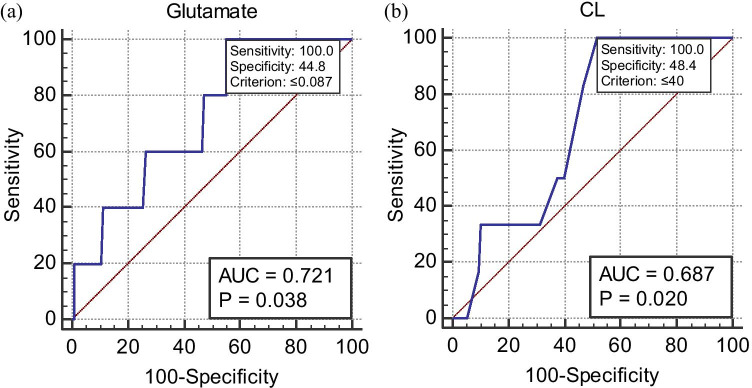
Receiver operating characteristic curve analysis of the performance of (**a**) cervicovaginal glutamate concentration and (**b**) cervical length (CL) for the prediction of spontaneous preterm birth in asymptomatic low-risk women at mid-gestation. *AUC*, area under receiver operating characteristic curve

**Fig. 2:**
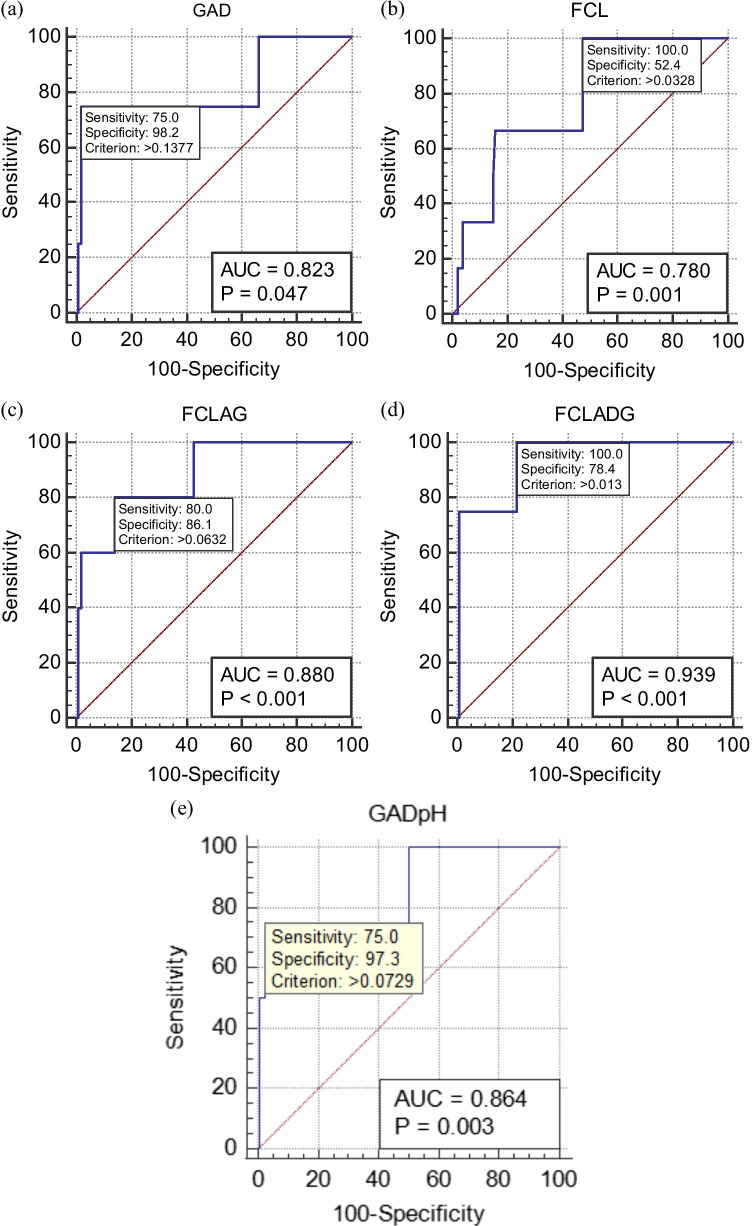
Receiver operating characteristic curve analysis of the performance of multivariable models for the prediction of spontaneous PTB in asymptomatic low-risk women at mid-gestation. a Glutamate, acetate and D-lactate (GAD). **b** Cervical length and quantitative fetal fibronectin (FCL). **c** Cervical length, quantitative fetal fibronectin, glutamate and acetate (FCLAG). **d** Cervical length, quantitative fetal fibronectin and GAD (FCLADG). **e** Glutamate, acetate, D-lactate and pH (GADpH). *AUC*, area under receiver operating characteristic curve

There were no significant differences in CVF metabolite concentrations between women with preterm and term delivery except the L/D-lactate ratio, which was significantly higher in the women that delivered at term (*P* = 0.04) (Supplementary Table 1). Individually, only glutamate (AUC = 0.72, 95% CI = 0.64–0.80) and CL (AUC 0.69, 95% CI = 0.60–0.77) were predictive of sPTB (Fig. 1 and Table 3). However, five multivariable models that more accurately predicted sPTB were also identified. The first model was a combination of glutamate, acetate and D-lactate (GAD), the second consisted of CL and qfFN only, the third consisted of CL, qfFN, glutamate and acetate, the fourth was a combination of CL, qfFN and GAD, while the fifth was a combination of glutamate, acetate, D-lactate and pH (Table 3 and Fig. 2).

**Table 3:** Predictive models for spontaneous preterm birth in asymptomatic low-risk women

Predictive models	AUC (95% CI)	Sensitivity (%)	False-positive rate (%)	Positive predictive value (%)	Negative predictive value (%)	Positive likelihood ratio	Negative likelihood ratio
Glutamate	0.72 (0.64–0.80)	100	55.2	6.8	100	1.8	0.0
CL	0.69 (0.60–0.77)	100	51.6	8.3	100	1.9	0.0
CL + qfFN	0.78 (0.70–0.85)	100	47.6	9.1	100	2.1	0.0
Glutamate + Acetate + D-lactate (GAD)	0.82 (0.74–0.89)	75	1.8	60	99.1	42.4	0.3
Glutamate + Acetate + D-lactate + pH (GADpH)	0.86 (0.79–0.92)	75	2.7	50.0	99.1	28.0	0.3
CL + qfFN + Glutamate + Acetate	0.88 (0.81–0.93)	80	13.9	20	99	5.8	0.2
CL + qfFN + GAD	0.94 (0.88–0.98)	100	21.6	14.3	100	4.6	0.0

*AUC*, area under receiver operating characteristic curve; *CI*, confidence interval; *CL*, cervical length; *qfFN*, quantitative fetal fibronectin

False-positive rate (100 – specificity)

Furthermore, associations between maternal clinical characteristics and CVF metabolite concentrations were observed. The Spearman’s correlation coefficient *r* and *P* values of the associations are listed in Table 4. Notably, vaginal pH negatively correlated with lactate and glutamate, but positively with acetate and formate. Similarly, qfFN negatively correlated with lactate though to a weaker extent. However, unlike vaginal pH and qfFN, CL positively correlated with glutamate. There were also correlations between glutamate and qfFN—negative—and lactate and CL—positive— showing trends toward significance (Table 4).

**Table 4:** Correlation of CVF metabolite concentrations and maternal clinical characteristics

	Lactate, g/L	Glutamate, g/L	Acetate, g/L	Formate, g/L
Vaginal pH	− 0.52, < 0.0001	− 0.40, < 0.0001	0.20, 0.03	0.27, 0.002
qfFN, ng/mL	− 0.27, 0.002	− 0.16, 0.08^a^	0.13, 0.16	0.10, 0.27
CL, mm	0.16, 0.06^a^	0.20, 0.03	− 0.12, 0.23	0.02, 0.82

Values are represented as Spearman’s correlation coefficient *r*, and probability *P*

*CL*, cervical length; *qfFN*, quantitative fetal fibronectin

^a^*P* values showing a trend toward significance

## Discussion

Identification of the underlying risk factor(s) driving the biological mechanism of sPTB is necessary for accurate selection of women for targeted interventions to prevent sPTB. This is perhaps more crucial in asymptomatic low-risk pregnancies where known risk factors may explain only a limited fraction of the incidence of Sptb [31]. We show, perhaps for the first time, the ability of a combination of metabolites (glutamate, acetate and D-lactate) produced by host-microbial interactions in the cervicovaginal space to identify pregnancies destined to end prematurely in an asymptomatic low-risk population. The CVF metabolite multivariable predictive model was more specific for sPTB with a positive predictive value (PPV) that was 6.6 times that of the combination of transvaginal ultrasound CL and qfFN. The metabolites also showed moderate correlations with vaginal pH, CL and qfFN. Larger studies are required to validate the clinical utility of this metabolite model to predict sPTB in asymptomatic low-risk pregnancies either as a unique test or in combination with current diagnostic strategies or other risk factors associated with sPTB in this cohort.

Expectedly, we observed a low (4.4%) incidence of sPTB in our study cohort, which is similar to other studies that reported rates between 3.0 and 7.3% in similar low-risk populations [19, 21, 22, 31, 32]. A study recorded an incidence rate of 7.3% but could not identify any marker—CVF IL-6 and IGFBP-1 as well as serum alkaline phosphatase, G-CSF, alpha-fetoprotein (AFP), IL-6 and ferritin—with useful predictive capacity [32].

The poor predictive abilities of transvaginal sonographic assessment of CL, cervicovaginal qfFN and other biochemical markers for sPTB in asymptomatic low-risk women indicate the need for evaluation of other screening tests. Although PTB is a multifactorial syndrome, there is substantial evidence supporting the role of ascending genital tract infection and inflammation in more than 50% of sPTB [33–35]. In our comparatively small study population, we observed that the combination of cervicovaginal glutamate, acetate and D-lactate predicted the risk of sPTB with high sensitivity, specificity, PPV and positive likelihood ratio. In fact, this model showed the lowest false-positive rate (1.8%) compared to models incorporating CL, qfFN and pH. An ideal screening test should be highly sensitive and specific, readily accessible, easy to perform, reproducible and accurate [32]. More importantly, such test should be able to guide bespoke and effective intervention to prevent or mitigate the incidence of sPTB.

D-lactate, glutamate and acetate are markers of microbial composition of the cervicovaginal space. An increase in D-lactate and glutamate is associated with eubiosis, while an increase in acetate is associated with dysbiosis and infection [23]. Increased lactate is associated with term delivery [24], whereas elevated acetate is associated with PTB with an ability to predict sPTB in symptomatic women [25–27]. This predictive capacity is improved by incorporating increased acetate with reduced lactate and glutamate, and increased production of IL-6 and TNFr-1 [27]. Lactic acid is predominantly produced by health-promoting *Lactobacillus* species, while acetate is mostly produced by anaerobic bacteria that cause opportunistic infections leading to adverse reproductive outcomes including sPTB [23, 36]. Elevated or intact glutamate concentration, often observed in healthy women, is indicative of *Lactobacillus* dominance [36], as lactobacilli do not metabolise amino acids for energy production [37].

This initial report on women without an established risk of preterm birth is limited by a small sample size. Larger sufficiently powered studies, with more women eventually delivering preterm, will be required to determine the potential clinical utility of this predictive multivariate model in low risk women. Meanwhile, we show that in asymptomatic pregnant women without a prior history of PTB, in whom CL and qfFN are usually ineffective in screening for risk of sPTB, CVF D-lactate, glutamate and acetate together is highly predictive and can be employed in screening low risk women.

Though moderately, the metabolites also correlated with vaginal pH. Vaginal acidity increased with increase in both lactate and glutamate, supporting their role in maintaining eubiosis. On the other hand, vaginal acidity reduced with increasing acetate and formate. Reduced vaginal acidity creates a conducive environment for potentially pathogenic anaerobes to thrive and cause infection such as bacterial vaginosis [38], a known risk factor for sPTB [23, 39]. Surprisingly, glutamate moderately increased in proportion to CL. Lactate showed a similar but non-significant trend, and decreased significantly with increasing qfFN. Glutamate also showed a non-significant decrease with increase in qfFN. We hypothesise that a dysbiotic vaginal milieu with decreased lactate and glutamate but elevated acetate may increase vaginal pH, induce cervical remodelling and fetal membrane activation as early as mid-trimester that can persist undetected and culminate in sPTB. These associations further highlight the possible, though destructive synergism between multiple risk factors in the pathogenesis of sPTB and warrants further exploration.

The present study has several limitations. Although the reported incidence rate of sPTB was similar to other larger studies, the interpretation of our data is limited by the relatively small sample size. Our study population was also predominantly Caucasians living in a high resource setting. This may have undermined the influence of race/ethnicity, environment and other sociodemographic factors on the risk of PTB and significantly hampered the generalizability of our findings. Larger multicentre studies with more sPTBs are required to validate the clinical applicability of CVF D-lactate, acetate and glutamate for screening of asymptomatic low-risk women at mid-gestation, and determine the interventions women with positive tests should benefit from. If our findings are confirmed in larger studies, clinical application of such test should be more accurate, readily accessible, easily performed, reproducible and cost-effective.

## Conclusion

In this cohort of asymptomatic low-risk women, a mid-trimester combination of CVF glutamate, acetate and D-lactate predicted preterm birth more accurately than individual metabolites, transvaginal ultrasound cervical length and quantitative fetal fibronectin with a very low false-positive rate and high positive predictive value. Further testing in a population with higher preterm birth rates is required.

## Supplementary Information

Below is the link to the electronic supplementary material.

Supplementary file1 (PDF 12 KB)
