# Supplementary material for: A Combination of Cervicovaginal Fluid Glutamate, Acetate and D-Lactate Identified Asymptomatic Low-Risk Women Destined to Deliver Preterm: a Prospective Cohort Study
Source: Reprod Sci. 2021 Aug 10;29(3):915–22. doi: 10.1007/s43032-021-00711-2 (PMC8863700; doi:10.1007/s43032-021-00711-2)
Supplement: Supplementary file 1 — Supplementary file1 (PDF 12 KB) [file 43032_2021_711_MOESM1_ESM.pdf]

Supplementary Table 1. Cervicovaginal fluid metabolite concentrations

**Metabolite, g/L**	**Term (N = 129)**	**Preterm (N = 6)**	***P* value**
Acetate	0.04±0.04 (n=121)	0.05±0.06 (n=6)	0.46
Glucose	0.58±0.56 (n = 129)	0.64±0.37 (n=6)	0.39
Glutamate	0.09±0.06 (n = 125)	0.05±0.03 (n=5)	0.10
Formate	0.01±0.01 (n=121)	0.01±0.01 (n=6)	0.67
D-lactate	0.31±0.38 (n=122)	0.30±0.25 (n=5)	0.96
L-lactate	0.39±0.25 (n=125)	0.33±0.30 (n=6)	0.75
Total lactate	0.68±0.46 (n=128)	0.58±0.32 (n=6)	0.71
L/D-lacate ratio	25.30±62.88 (n=113)	0.44±0.38 (n=3)	**0.04**
Succinate	0.03±0.03 (n=70)	0.03±0.03 (n=5)	0.85
Acetate/lactate ratio	0.16±0.51 (n=118)	0.43±0.94 (n=6)	0.50
Succinate/lactate ratio	0.05±0.06 (n=69)	0.26±0.53 (n=5)	0.75

Data are presented as Mean ± Standard deviation. Reduced sample numbers (n) in some metabolites are due to concentrations below the detectable limit of the assay kit.
